# Better Language — Faster Helper: The Relation Between Spontaneous Instrumental Helping Action and Language Ability in Family-Reared and Institutionalized Toddlers

**DOI:** 10.11621/pir.2021.0406

**Published:** 2021-02-12

**Authors:** Olga Kochukhova, Yulia Dyagileva, Anna Mikhailova, Lilia Orekhova, Sergei Makhin, Vladimir Pavlenko

**Affiliations:** a Department of Psychology, Department of Women’s and Children’s Health, Uppsala University, Sweden; b V.I. Vernadsky Crimean Federal University, Simferopol, Russian Federation

**Keywords:** Toddlers, institutional rearing, prosocial behavior, instrumental helping, language skills

## Abstract

**Background:**

Prosocial behavior is the key component of social and interpersonal relations. One of the elements of prosociality is helping behavior, which emerges already in early childhood. Researchers have identified several domains of helping behavior: instrumental helping, comforting another person, and sharing resources with others. The development of helping behavior can depend on a number of factors: children’s age, the social situation of development, communication skills, and the ability to understand the feelings and needs of another person.

**Objective:**

In Study 1, the main goal was to determine the effects of age and cognitive, language, and motor development on instrumental helping skills in early childhood. The goal of Study 2 was to estimate the effects of rearing in an adverse social environment by comparing the capacity for instrumental helping in family-raised and institutionalized children.

**Design:**

The authors examined toddlers’ (N = 198) ability to initiate spontaneous helping and the factors that may influence it. Cognitive, language, and fine motor skills were measured by the Bayley Scales of Infant and Child Development, 3rd edition. Children’s instrumental helping behavior was assessed according to the procedure presented by Warneken and Tomasello, with a few modifications.

**Results:**

Study 1 demonstrated that children’s ability to initiate helping was dependent on their age: the non-helpers were significantly younger than the helpers. Children’s language skills also played a significant role in their helping behavior. The children with higher language skills helped the adult more often and more quickly. Study 2 demonstrated that institutional placement per se was not related to toddlers’ ability to initiate helping. Language ability was associated with helping behavior both in institution- and family-reared toddlers.

**Conclusion:**

Instrumental helping in early childhood is related to children’s age, language skills, and rearing conditions.

## Introduction

Prosocial behavior has been demonstrated to be an important aspect in people’s lives in different populations (Inglehart et al., 2014; Köster, & Kärtner, 2019). According to Eisenberg, Fabes, and Spinrad (2006), prosocial behavior can be defined as voluntary action that is performed in response to others’ needs and is intended to benefit others. Dunfield and colleagues (Dunfield, 2014; Dunfield & Kuhmeier, 2013; Dunfield, Kuhlmeier, O’Connell, & Kelley, 2011) developed this definition by proposing that prosocial behavior includes at least three specific domains focused on responding to different needs of others: instrumental helping as a response to others’ inability to finish a specific goal-directed action; comforting as the ability to respond to others’ emotional needs; and sharing as a response to others’ lack of a desired material need. This study will focus on development of the instrumental helping domain. license.

### Origins of Prosocial Behavior and Factors Affecting It

Warneken and Tomasello (2009) argued for an innate nature of prosocial behavior. In their opinion, human infants have a phylogenetic predisposition to help others. Taking this into account, we would expect that helping behavior should appear early in development and independently of the environment in which the child lives, as part of children’s natural maturation process. In other words, the childs age should be a strong predictor of the ability. At the same time, prosocial behaviors are important components of the child’s social functioning and are encouraged by human society (Warneken, Hare, Melis, Hanus, & Tomasello, 2007). Other research has demonstrated that prosocial behaviors develop early (Over & Carpenter, 2009; Paulus & Moore 2012; Svetlova, Nichols, & Brownell, 2010; Warneken & Tomasello, 2006), different domains develop quite independently of each other (Dunfield & Kuhmeier, 2013), and instrumental helping behavior is the first to emerge in one’s development (Dunfield, 2014; Svetlova et al., 2010). Evidence for environmental influence on the development of instrumental helping behavior is somewhat contradictory. An early appearance of instrumental helping ability suggests some phylogenetical component in its development. In line with this, Brownell and colleagues (Brownell, Svetlova, Anderson, Nichols, & Drummond, 2013) demonstrated that parental reading of fairy tales and discussing the characters’ emotions with their children did not affect the children’s capacity for instrumental helping, but enhanced their comforting behavior. On the other hand, several studies have indicated that children’s instrumental helping is influenced by their social motives and previous experience (Dunfield & Kuhlmeier, 2010; Kärtner, Schuhmacher, & Collard, 2014; Köster, Cavalcante, Vera Cruz de Carvalho, Dôgo Resende, & Kärtner, 2016; Over & Carpenter, 2009).

According to Köster and colleagues (Köster, Itakura, Omori, & Kärtner, 2019), emergence of instrumental helping during the second year of life becomes possible as the child’s fine motor skills and social interactions continue to develop. Indeed, before helping behavior can take place, a child must understand that the other person is in need of help, and the child’s motor system should be sufficiently developed to carry out the helping action. This idea was supported by Warneken and Tomasello (2006, 2007), who noted that 14-month-old toddlers are already developed enough not only to understand when a person needs help to reach her goal, but also to perform instrumental helping in different situations. Moreover, the appearance of instrumental helping behavior coincides in time with the emergence of speaking ability, a scaffold-ing instrument for social interactions. Most children start to talk in about the second half of the second year, although understanding of speech appears earlier. Thus, it can also be assumed that individual differences in language, motor, and cognitive skills can influence the development of instrumental helping behavior (Cassidy, Werner, Rourke, Zubernis, & Balaraman, 2003; Ensor & Hughes, 2005).

Previous research gives the impression that social context is important mostly for the development of children’s comforting behavior. Results are less convincing in the case of the earliest form of prosocial behavior— instrumental helping— assuming a weak influence of social environment on its development. On the other hand, environmental influence may depend on how adverse the environment is. Previous research explored the development of instrumental helping ability in multicultural contexts where all situations had one important thing in common: The children were involved in meaningful social interactions with their caregivers (Dahl, 2015; Dahl et al., 2017; Köster et al., 2016). However, there are situations where children are deprived of this opportunity, as for example, when growing up in adverse environments of institutional rearing.

### Influence of Institutional Rearing on Children’s Cognitive and Social Development

Institutional rearing remains the main alternative for child-care of orphans in many developing countries (Browne, 2005). It often implies a high children-to-caregiver ratio, frequent changes and multiple shifts of caregivers, in combination with highly regimented care (Dobrova-Krol, Van Ijzendoorn, Bakermans-Kranenburg, Cyr, & Juffer, 2008). Furthermore, the caregivers typically demonstrate low emotional engagement when interacting with children (McCall, Van Ijzendoorn, Juffer, Groark, & Groza, 2012).

There is a growing body of research that demonstrates abnormal neural development in young children living in an adverse environment of institutional rearing (Belalov, Dyagileva, Pavlenko, & Kochukhova, 2014; Kochukhova, Mikhailova, Dyagileva, Makhin, & Pavlenko, 2016; McLaughlin, Sheridan, & Lambert, 2014; Nelson, Bos, Gunnar, & Sonuga-Barke, 2012; Nelson & Gabard-Durnam, 2020; Smyke et al., 2007; Stamoulis, Vanderwert, Zeanah, Fox, & Nelson, 2017). For example, Sheridan and colleagues (Sheridan, Fox, Zeanah, McLaughlin, & Nelson, 2012) revealed that the reduction of resting EEG g-power is partly mediated by a general reduction of cortical white matter volume in Romanian institutionalized children. The white matter reduction implies fewer properly working connections between different brain areas engaged in information processing. On the behavioral level, these brain alterations are reflected in various developmental deviations. That, in turn, can be one of the factors that influence children’s ability to provide help to other people, by both delaying the ability and by slowing the children’s helping response.

In our previous studies, we evaluated the cognitive, language, and motor development in young children raised in families and compared their scores to children of the same age raised in an orphanage (Belalov et al., 2014, Belalov et al., 2017). The orphanage-reared children demonstrated lower scores in all measured domains. To our knowledge, this was the only study in which the developmental status of children living in the Republic of Crimea was estimated by means of the Bayley Scales, which are known for their accurate measurement.

To sum up, based on the earlier research it can be suggested that the emergence of instrumental helping behavior can be influenced by several factors. One is children’s general maturation process, as well as their individual characteristics in cognitive, language, and motor skills. On the other hand, the environment in which the children are raised can also be important.

#### Main goals of the study

We conducted two studies aimed at evaluating different factors that may influence children’s helping behavior at an early age. In Study 1, we explored whether children’s age or their test scores in cognitive, language, and motor skills could predict their ability to perform instrumental helping. In Study 2, we compared instrumental helping behaviors between children raised in their biological families and those reared in an orphanage, in regard to their cognitive and language development.

#### Hypothesis

The emergence of instrumental helping behavior in early childhood can be influenced by children’s age, their cognitive, language, and motor skills, as well as the social environment in which they are raised.

## Methods

### Participants

Children from the family-reared (FR) group were recruited through announcements in kindergartens in Simferopol, Crimea. The FR children comprised 100 subjects (53girls), aged between 259 and 1,113 days (mean age = 802 ± 207 days). All the parents stated that their children lived in two-parent families, and none of them had any history of institutionalization. Ninety of them were of Russian or Ukrainian ethnicity (Slavs) and 10 were Crimean Tatars. In the Crimean Tatar families, the parents reported that Russian was the main language of communication. In 69% of the families, at least one parent had a higher education diploma. At least one parent in each family had a full-time job. All the parents estimated their earnings as average for the region.

Children from the institution-reared (IR) group were recruited from a child residential care institution in Simferopol, Crimea. The group consisted of 49 toddlers aged between 650 and 1,256 days (15 girls, mean age = 1,015 ± 165 days). All participants from the institution-reared group had lived on a permanent basis in the child residential care facility since admission and had spent there between 47 and 1,143days (3.4–95% of their lives; *M* = 438, *SD* = 303 days). The IR sample consisted of 46 children with Russian or Ukrainian ethnicity (Slavs); three children were Crimean Tatars.

Children were included in the study according to the following criteria: no genetic syndromes (e.g., Down syndrome), no expressed signs of fetal alcohol syndrome, no HIV infection, cerebral palsy or chronic diseases, birth weight not less than 2,500 g, and right hand preference when drawing and eating.

### Measures

#### Cognitive, Language, and Fine Motor Development

Cognitive, language, and fine motor abilities were assessed using the Bayley Scales of Infant and Child Development, third edition (BSID-III) (Bayley, 2006). BSID-III is developed for an age range of 1–42 months. The cognitive scale of development consists of items assessing various abilities, such as puzzle completion, search for hidden objects, imitation, comparison, elimination of irrelevant items, memorization, and understanding of cause-and-effect patterns. The Language Index is calculated as the average between the scores in Expressive and Receptive (auditory comprehension) language skills. It includes tasks aimed at assessing understanding and use of names of objects, verbs, pronouns, participles, past, present, and future tenses, synonyms and antonyms, understanding of colors, “parts and the whole”, size, etc. The fine motor scale assesses skills such as grasping, stacking blocks, drawing, lacing, and cutting with scissors. Each scale consists of a different number of tasks, organized into 17 blocks, ascending in difficulty. Each block corresponds to a specific age. Before testing, the child’s age in months and days is calculated. The test starts at the block of tasks appropriate to the child’s age. If the child fails to complete the first three items in this block, testing restarts with the items in the previous block. The testing stops if the child fails to complete five tasks in a row. All tasks are conducted in the form of a game.

#### Instrumental Helping

**Figure 1.**
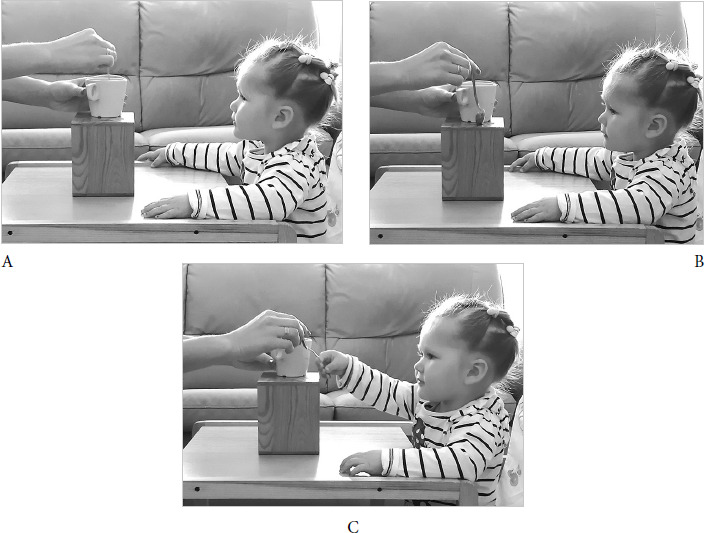
Experimental setup for assessing instrumental helping behavior in children. A. The experimenter places a teacup on the box and stirs the “tea” with a teaspoon. B. The experimenter accidentally drops the spoon through a narrow hole on the top of the box. C. The child hands the spoon to the experimenter

Children’s instrumental helping behavior was assessed according to the procedure presented by Warneken and Tomasello (2006), with some modifications. The child was placed on one side of a table, sitting opposite the experimenter. A box was placed in front of the child, with a narrow hole at the top and an open side directed toward the child (see *Figure 1*). Thus, the child was able to see what is in the box and could easily get objects out of it. The experimenter said that she needs to prepare some tea and went to another table in order “to make it”. She came back with a teacup and placed it on the box, where she continued “to stir the tea” with a spoon and “accidentally” dropped the spoon into the narrow hole in the box. Without looking at or saying anything to the child, she unsuccessfully tried to reach the spoon through the narrow hole. After 9–17 seconds (mean time = 13.3 s, *SD* = 2), if the child did not return the spoon, the experimenter said, “I dropped the spoon”, looked at the child, and continued to try to pick it up. If the child still did not return the spoon, the same procedure was repeated twice more.

We registered whether the child returned the spoon and measured the time delay before the child initiated the returning action. All of the children either returned the spoon during the first minute after “the accident” or did not return it at all. We estimated the time that passed before the child returned the spoon by examining the video records.

## Procedure

Each child was tested individually. Two experimenters collected the data during two consecutive days.

### Day one

A child came into the testing room where he/she could play freely with some toys to get used to the environment. Then the child was seated either on the caregiver’s lap or independently on a chair in front of a table. After that, assessment with the BSID-III cognitive scale was performed. Then the experimenter said that she would like to drink some tea and the helping behavior task started. The entire session with all pauses lasted about 30–60 minutes.

### Day two

The testing session started with free play, and then the BSID-III language and fine motor scales were performed. The session took 30–40 minutes.

All family-reared children were tested in the presence of a parent or close relative in a testing room at the university campus. Institution-reared children were tested in the presence of the residential care psychologist in a separate room at the residential care institution.

Out of 100 children invited to participate in Study 1, one was excluded from further analysis because of the parent’s interference during the helping behavior test. Language development scales were completed only for 86 children (eight children did not appear on the second day of the study; six lost interest during testing). In Study 2, two out of 49 FR children did not complete both language scales. Out of 49 IR children, five did not participate as they were absent from the orphanage for medical reasons or because of participation in cultural events. The actual numbers of children included in certain statistical analyses can be inferred by the degrees of freedom indicated, together with the calculated statistical coefficients.

### Statistical Processing

The data were statistically processed using IBM SPSS Statistics v.24.

#### Study 1

Spearman rank correlations were calculated to measure the associations between age and cognitive, language, and motor skills, on the one hand, and helping action initiation and delay, on the other. T-tests were used to compare the Bayley scores of helpers and non-helpers.

Logistic regression analysis was performed to assess the predicting power of the variables: children’s age and cognitive, language, and motor abilities for prediction of helping action. The equality of medians between children returning the spoon before and after the experimenter’s phrase “I dropped the spoon” was tested using the Mann-Whitney U test.

#### Study 2

Pearson correlations were used to estimate the association between delay in helping and cognitive and language ability, separately for the FR and IR groups. T-tests were used to compare the Bayley scores of the FR and IR groups.

Logistic regression analysis was also performed to ascertain the effects of group affiliation and language ability on the likelihood that participants would perform a helping action. The equality of means of children returning the spoon before and after the experimenter’s phrase “I dropped the spoon” was tested with the Mann-Whitney U test.

One-way ANOVA was used to estimate the effect of group affiliation (FR vs. IR) on the children’s cognitive and language development.

## Results

### Study 1

The first aim of Study 1 was to explore the associations between the children’s age and cognitive, language, and motor ability, and their ability to demonstrate instrumental helping behavior. The second aim was to evaluate the level of spontaneity in children’s helping actions and whether it was associated with their age and cognitive, language, and fine motor abilities. Only family-reared children participated in Study 1.

On the Bayley scales, the children showed an estimated average value of 12.1 (*SD* = 2.5) points on the Cognitive scale, 11.5 (*SD* = 2) on the Language Index, and 12.8 (*SD* = 3.1) on the Fine Motor scale.

Eleven out of 100 children did not help at all. The non-helpers were significantly younger than the helpers (643 ± 206 days vs. 819 ± 201 days, *t* (97) = –2.6, *p* < 01, *d* = .87) and had lower language ability for their age (8.8 ± 2.25 vs. 11.7 ± 1.97, *t*(84) = –2.6, *p* < .001, *d* = 1.45; 95% *CI*: 0.79, 2.11). After the spoon was dropped, it took on average of 12.0 s (*SD* = 11) for children to initiate helping. In general, the ability to initiate helping was significantly related to the children’s age (*r_s_*(99) = .25, *p* < .01) and level of language development (*r_s_*(86) =.29, *p* < .01). In order to explore which of these factors can predict helping action, we performed logistic regression analysis. The logistic regression model was statistically significant, *X^2^*(2) = 15.8, *p* < .001. The model explained 42.3% (Nagelkerke *R^2^*) of the variance in helping behavior and correctly classified 95.3% of cases. Only better language ability was associated with an increased likelihood of helping action (*OR* = 1.94, *p* =.02, *d* = .37; 95% *CI*: 1.11, 3.37).

In order to explore which factors are associated with the time delay before helping was initiated, we conducted correlation analysis between helping time delay and children’s age and cognitive, language, and fine motor development. The results demonstrated that only the level of language development was associated with helping time delay, *r_s_*(79) = –.26, *p* < .02.

During the helping trial procedure, in order to draw the child’s attention to the task, the experimenter uttered the phrase “I dropped the spoon”. We assumed that the association between helping delay, as well as helping action itself and the level of language development, could be influenced by the utterance. In order to check this, we performed additional analyses. Eighty-nine children returned the spoon to the experimenter. Sixty-three of them returned the spoon before the utterance took place. This subgroup did not differ in any measured variables from the children who returned the spoon after the utterance, except that the before-group had higher language ability (*Mdn* = 12.5 vs. *Mdn* = 10.5, *U* = 374, *p* = .031, *d* = .94).

### Study 2

The second study had two purposes: First, we intended to explore whether an adverse environment of institution placement can be associated with children’s helping behavior patterns, and second, to find out whether the results of Study 1 for family-reared children can be replicated. We presented 98 children aged 662–1,281 days with the same experimental setting as in Study 1. The participants comprised two groups: children residing at an institution and children living in two-parent families. All the children were old enough (according to previous studies) to perform helping action in situations even more complex than the one we presented. Based on this and on the results obtained in Study 1, we expected no significant influence of fine motor skills on children’s helping behavior. Hence, we decided not to include fine motor skills measurement in the Study 2 design. The final sample of the family-reared (FR) group consisted of 49 toddlers aged 661–1,261 days (15 girls, mean age = 1021 ± 171 days). They were matched to the IR children for age and sex, differing in age by no more than 15 days. The description of the IR group is given in the Methods section.

After the spoon was dropped, it took an average of 13.3 s (*SD* = 13.4) for FR children to initiate helping. Only two children (4.1%) did not return the spoon. The institution-reared group demonstrated comparable time results with an average time of 15.5 s (*SD* = 15), *t* (84) = .75, *d* = -.16, *n.s*. Ten of the 49 children (20.4%) in this group did not return the spoon, which was significantly more in comparison to the FR group, *X^2^*(1, 98) = 9.5, *p* < .002, *d* = .65.

IR children performed significantly lower on the cognitive and language indices on the BSID-III scales compared to the family-reared group (*F*(1,78) = 38.5, *p* < .001, *ɳp^2^* = .33); mean cognitive development scores for IR (9.2 ± 1.6) vs. for FR (11.1 ± 2.4) group, *t*(93) = –4.7, *p* < .001 *d* = .93; language index scores: 8.3 ± 1.5 vs. 11.4 ± 2.5, correspondingly, *t*(81) = –6.7, *p* < .001, *d* = 1.95, Bonferroni-corrected.

The subgroup of IR children who did not return the spoon had a cognitive ability comparable to the IR children who did return it (9.7 ± 1.9 vs. 9 ± 1.5), but their language ability was significantly lower (6.86 ± 1.0 vs. 8.65 ± 1.4, *X^2^*(1) = 10.2, *p* < .001, *d* = .70).

A logistic regression was performed to ascertain the effects of group affiliation and language ability on the likelihood that participants would perform helping action. The regression model was statistically significant, *X^2^*(2) = 17.1, *p* < .001, explained 39.6% (Nagelkerke *R^2^*) of the variance in initiation of helping action and correctly classified 91.6% of observed cases. The only significant predictor of helping action was the children’s language ability (*OR* = 2.593, *p* < .01; *d* = .53; 95% *CI*: 1.29 5.20). Better language ability was associated with increased likelihood of helping.

In order to explore whether the children’s cognitive ability, language development, or group affiliation were associated with how spontaneous the helping action was, we performed correlation analyses. The results demonstrated that only the level of language development was associated with a delay in helping in both groups of children (IR: *r* (31) = –.43, *p* < .02; FR: *r* (43) = –.31, *p* < .05). In other words, the better the language ability was, the less time it took for the children to initiate the helping action, and the more spontaneous they were.

The second study used the same procedure as Study 1. The experimenter attracted the child’s attention with the phrase, “I dropped the spoon”. When we compared the family-reared children who returned the spoon before and affer the phrase was spoken, we could see that the only significant difference between these two subgroups was their language ability (*U* = 84.5, *p* < .002). The children who returned the spoon before the phrase was spoken had higher language ability (*Mdn* = 12.5 vs. *Mdn* = 10). The same analysis was performed on the institution-reared group. The results did not demonstrate significant differences between children in the before- and after-phrase subgroups (language ability, *Mdn* = 9 vs. *Mdn* = 8, *U* = 112, *p* = .12).

## Discussion

Study 1 demonstrated that the ability to initiate a helping action is partially linked to children’s maturational process. The non-helpers were significantly younger than the helpers. Language ability also played a significant role in the initiation of helping. The children with higher language development had a proclivity to help the experimenter more often and more quickly.

Other authors (Ensor and Hughes, 2005) indicate that, according to parents, toddlers who are better at speaking volunteer more often to help others (parents, teachers, other children). In preschoolers (37–65 months of age), greater language ability was related to almost all positive social behaviors as rated by teachers, observers, and other children (Cassidy et al., 2003).

This can be interpreted in different ways. According to Köster and Kärtner (2019), early helping emerges in the context of social interaction, which includes processes of social learning. Thus, we can assume that the association between language development and instrumental helping is greatly underpinned by the role of language in the social interaction between a child and other people.

This line of reasoning is confirmed by the study of Dahl (2015), who demonstrated that more frequent encouragement and social reinforcement of helping behaviors in one-year-old children made them more inclined to help their parents later on. A similar pattern was also demonstrated in a laboratory setting. The experimenter’s explicit scaffolding of helping behaviors (encouragement and praise) in 13–18-month-old children at the beginning of the study resulted in a significant increase of their instrumental helping reactions later on (Dahl et al., 2017). Thus, a relatively high level of language ability for a certain age facilitates children’s perception of such encouragements.

It is interesting that the phrase “I dropped the spoon” provided additional stimulation to initiate helping action in children with a less developed language ability. By providing this phrase in the experimental setting, we planned to draw the child’s attention to the situation without giving any direct clues what should be done. According to Lev Vygotsky’s idea (Vygotsky, 1978), acquisition of language enables children to overcome impulsive actions and better control their behavior. Taking this in the context of the present study, it seems possible that children with better language skills were better able to follow the development of the situation. Other children could do the same with additional, spoken stimulation from the experimenter.

In Study 2, we demonstrated for the first time that instrumental helping behavior is significantly less developed in institution-reared toddlers than in family-reared ones. Significantly fewer IR children demonstrated helping action. Their helping behavior was not associated with the amount of time they had spent at the institution, supporting the idea that inability to initiate helping action was not related to the adverse environment of the institution per se.

It has already been noted that children raised in orphanages usually have a lower level of cognitive development (Berens and Nelson, 2015; Kolesnikova, Zhukova, & Ovchinnikova, 2018; Nelson, Zeanah, & Fox, 2019; van Ijzendoorn, Luijk, & Juffer, 2008) and delay in speech understanding and generation (Albers, Johnson, Hostetter, Iverson, & Miller, 1997; Belalov et al., 2014; Cermak & Daunhauer, 1997; Morison, Ames, & Chisholm, 1995; Windsor, Glaze, Koga, & Bucharest Early Intervention Project Core Group, 2007). The results of this study of IR children conform well with the previous research. Thus, the IR group might had a greater problem seeing the goal of the experimenter’s actions as she tried to retrieve the spoon, which is unreachable from her position; however, the level of cognitive development in the IR group, as well as in both FR groups, was not associated with an ability to demonstrate instrumental helping action. Considering that the ability to demonstrate helping action was linked to the children’s level of language development, it is possible that the IR children had difficulty understanding the situation because it was sketched with the help of several statements by the experimenter. First, the experimenter said that she needed to prepare some tea, and when the children did not return the teaspoon, she added the phrase “I dropped the spoon”. This explanation is also supported by the FR groups’ results from both studies. In both family-reared groups, the helpers had better language ability, and were also more spontaneous/quicker to initiate helping. The same tendency could be observed in the IR group. Although, the difference in language ability between quick helpers and those who helped after the task-attention phrase was uttered did not reach statistical significance, the language ability was somewhat higher in the before-phrase subgroup. It is possible that in the case of the IR children, we did not have enough statistical power. The number of children in the IR group who showed helping behavior was smaller than in the FR group (39 vs. 47). Further, the institution-reared children, in general, had a lower and a tighter range of language index scores (5.5–12 vs. 6–17 in family-reared children). So, these results can also assume that in order to be able to initiate spontaneous/quick helping action, language should be developed over some threshold level that helps the child to follow the dynamics of the situation. This idea is supported by the IR non-helpers’ characteristics. Their cognitive development was comparable to the helpers subgroup, but their language abilities were significantly lower, as was also observed in the FR non-helpers in Study 1.

The discovered link between language development and instrumental helping actions is not to be explained, in our opinion, merely by language understanding. Better language ability can be based on better developed mechanisms of joint attention, letting the children more effectively engage in collaborative activities with others. According to Tomasello and his colleagues (Gräfenhain, Carpenter, & Tomasello, 2013; Tomasello, 2008), engagement in such activities structured by joint attention directly relates to how fast children begin to acquire their first linguistic conventions. In an institution, where a small number of teachers usually supervise a large number of children, there is much less possibility for formation of an adequate shared space of action. This can explain both delayed language ability and difficulties with initiation of helping action in institutionalized children.

Insufficient development of language in IR children is often seen as one of the grounds for emergence of so-called quasi-autistic behavior (see review in Berens and Nelson, 2015). Such children tend to interact with others in an inadequate manner, often play in isolation or in parallel with one another (Daunhauer, Coster, Tickle- Degnen, & Cermak, 2010). As a result, they have an underdeveloped capacity for reciprocal interactions with each other of a contingent or cooperative sort, which can also influence their ability to initiate helping action. Moreover, the formation of a shared action space for institutionalized children interacting with adults is usually structured in such a way that the children’s actions are determined less by their own initiative than by the expectation of commands from adults. Based on the results of the present study, it is reasonable to conclude that in early childhood, motivation to help others may not be enough on its own for effective helping behavior. When language and/or shared action space are underdeveloped, children need some additional guidance. That is what we observed in both FR and IR children with lower language ability.

An important factor that may underlie the link between helping behavior and language ability is the close multidirectional connections between language development and theory of mind (ToM) development (de Villiers, 2007). Language ability facilitates development of psychological understanding and through this helps a child to develop his/her theory of mind (Cassidy et al., 2003; de Villiers, 2007; de Villiers, J. & de Villiers, P.A., 2014; Milligan, Astington, & Dack, 2007). The earliest stages of communication depend on the infant’s interest in and engagement with other social beings who possess minds of their own. It is through these interactions that children acquire knowledge of words and meanings. A regular practice of speaking with adults about other people’s feelings and emotions leads to a more developed ToM in children, which helps them to better comprehend social situations. Such comprehension is critically important for understanding the goals underlying others’ actions, in that supporting further helping behaviors.

We did not find any association between cognitive development and helping in all the groups of children. The occurrence of helping actions after the phrase is uttered that draws attention to the task suggests a possible connection between helping behavior, language development, and executive functions, namely attention. It is possible that the picture would be somewhat different if, instead of general cognitive ability, we had measured children’s attention. This idea is partly supported by the studies of institutionalized toddlers that demonstrated deviant EEG patterns when processing verbal information (Belalov et al., 2014) and during a visual attention task (Kulenkova, Dyagileva, Pavlenko, Belalov, & Kochukhova, 2015).

To sum up, the relation between language development and helping actions revealed in this study requires more detailed research. It would be informative to study the characteristics of children’s attention and joint attention ability and ToM development in relation to instrumental helping in different situations and also in relation to other prosocial behaviors.

## Conclusion

Toddlers’ ability to initiate instrumental helping is dependent on their age. The non-helpers were significantly younger than the helpers. The level of language development was significantly correlated with the capacity for instrumental helping in the family-reared group of children. The children with a higher level of language development had a proclivity to help the experimenter more often and more quickly. Institution-reared toddlers demonstrated less developed instrumental helping compared to family-reared ones. Institution-reared non-helpers showed less developed language skills compared to helpers.
